# *catena*-Poly[[[bis­(nitrato-κ*O*)copper(II)]-bis­[μ-1,3-bis­(imidazol-1-yl)-5-methyl­benzene-κ^2^ *N* ^3^:*N* ^3′^]] dihydrate]

**DOI:** 10.1107/S1600536812023628

**Published:** 2012-05-31

**Authors:** Guang-Xiang Liu

**Affiliations:** a School of Biochemical and Environmental Engineering, Nanjing Xiaozhuang University, Nanjing 211171, People’s Republic of China

## Abstract

In the title complex, {[Cu(NO_3_)_2_(C_13_H_12_N_4_)_2_]·2H_2_O}_*n*_, the Cu^II^ atom is located on a crystallographic center of symmetry and adopts an N_4_O_2_ octa­hedral coordination geometry with four imidazole N atoms in the equatorial sites and two O atoms in the axial sites. The dihedral angles between the central benzene ring and the imidazole rings are 4.93 (11) and 46.08 (12)°. The 1,3-bis­(imidazol-1-yl)-5-methyl­benzene ligand is bis-monodentate, linking symmetry-related Cu^II^ atoms into sheets in the *bc* plane. These sheets are further bridged into a three-dimensional supra­molecular structure by O—H⋯O and C—H⋯O hydrogen bonds.

## Related literature

For background to the coordination chemistry of imidazole derivates, see: Huang *et al.* (2006 ▶); Wang *et al.* (2008 ▶); Tian *et al.* (2007 ▶); Jin *et al.* (2008 ▶). For imidazole ligands bearing rigid spacers, see: Qi *et al.* (2008 ▶); Li *et al.* (2007 ▶); Zhang *et al.* (2008 ▶). For the synthesis, see: Altman & Buchwald (2006 ▶).
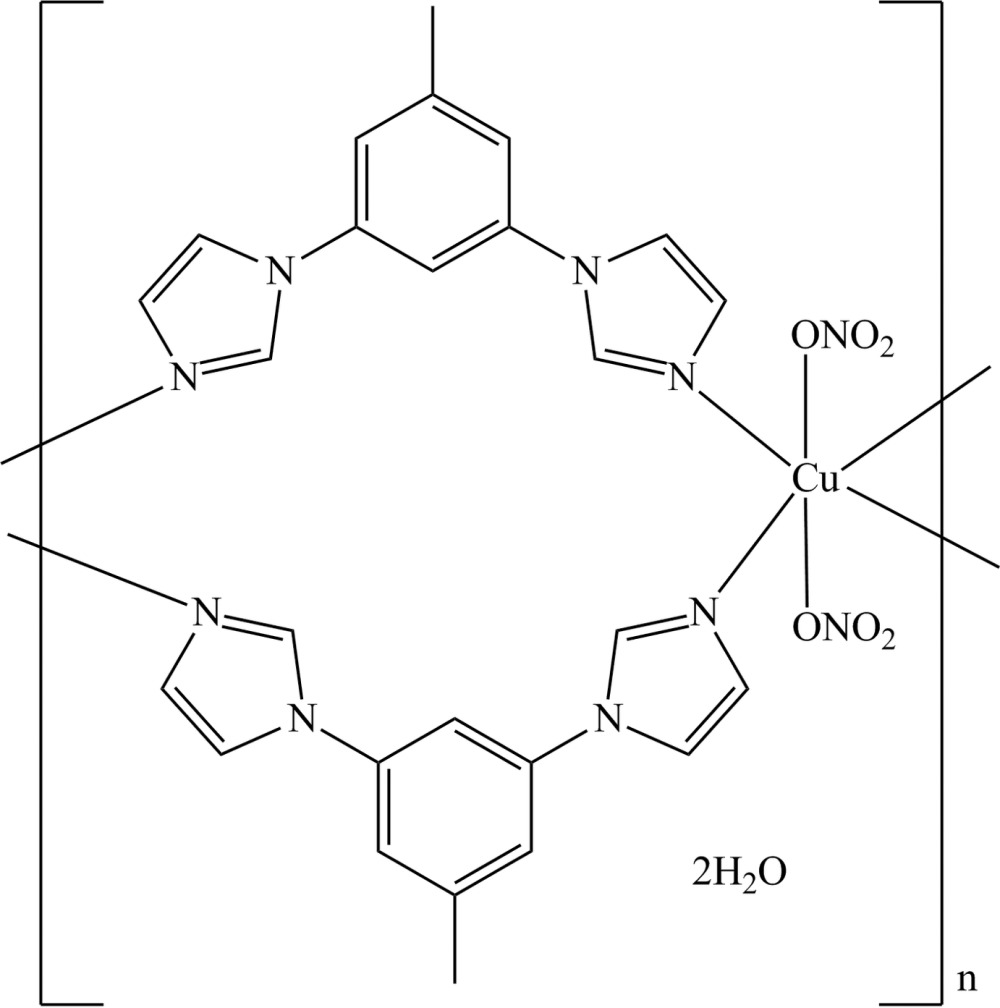


## Experimental

### 

#### Crystal data

[Cu(NO_3_)_2_(C_13_H_12_N_4_)_2_]·2H_2_O
*M* *_r_* = 672.12
Monoclinic, 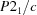

*a* = 11.585 (4) Å
*b* = 9.652 (3) Å
*c* = 15.450 (4) Å
β = 123.604 (17)°
*V* = 1438.9 (8) Å^3^
*Z* = 2
Mo *K*α radiation
μ = 0.83 mm^−1^
*T* = 293 K
0.22 × 0.20 × 0.18 mm

#### Data collection

Bruker SMART APEX CCD area-detector diffractometer
Absorption correction: multi-scan (*SADABS*; Bruker, 2000 ▶) *T*_min_ = 0.839, *T*_max_ = 0.865
10260 measured reflections
2672 independent reflections
2114 reflections with *I* > 2σ(*I*)
*R*_int_ = 0.039

#### Refinement

*R*[*F* ^2^ > 2σ(*F* ^2^)] = 0.036
*wR*(*F* ^2^) = 0.101
*S* = 1.06
2672 reflections
214 parameters
2 restraints
H atoms treated by a mixture of independent and constrained refinement
Δρ_max_ = 0.43 e Å^−3^
Δρ_min_ = −0.51 e Å^−3^

### 

Data collection: *SMART* (Bruker, 2000 ▶); cell refinement: *SAINT* (Bruker, 2000 ▶); data reduction: *SAINT*; program(s) used to solve structure: *SHELXS97* (Sheldrick, 2008 ▶); program(s) used to refine structure: *SHELXL97* (Sheldrick, 2008 ▶); molecular graphics: *SHELXTL* (Sheldrick, 2008 ▶); software used to prepare material for publication: *SHELXTL*.

## Supplementary Material

Crystal structure: contains datablock(s) I, global. DOI: 10.1107/S1600536812023628/lr2064sup1.cif

Structure factors: contains datablock(s) I. DOI: 10.1107/S1600536812023628/lr2064Isup2.hkl

Additional supplementary materials:  crystallographic information; 3D view; checkCIF report

## Figures and Tables

**Table 1:** Hydrogen-bond geometry (Å, °)

*D*—H⋯*A*	*D*—H	H⋯*A*	*D*⋯*A*	*D*—H⋯*A*
O1*W*—H1*WA*⋯O3	0.88 (2)	2.04 (2)	2.909 (6)	170 (4)
O1*W*—H1*WB*⋯O3^i^	0.86 (2)	2.20 (3)	3.020 (5)	159 (5)
O1*W*—H1*WB*⋯O2^i^	0.86 (2)	2.42 (4)	3.142 (4)	142 (5)
C2—H2⋯O1*W*^ii^	0.93	2.36	3.230 (5)	156
C3—H3⋯O1^iii^	0.93	2.27	3.186 (4)	167

Symmetry codes: (i) 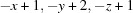; (ii) 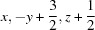; (iii) 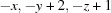.

**Fig. 1.**
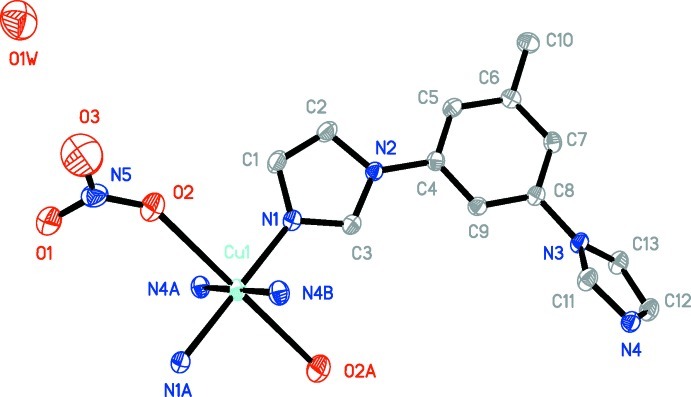
The title complex with displacement ellipsoids shown at the 30% probability level. Hydrogen atoms are omitted for clarity.

**Fig. 2.**
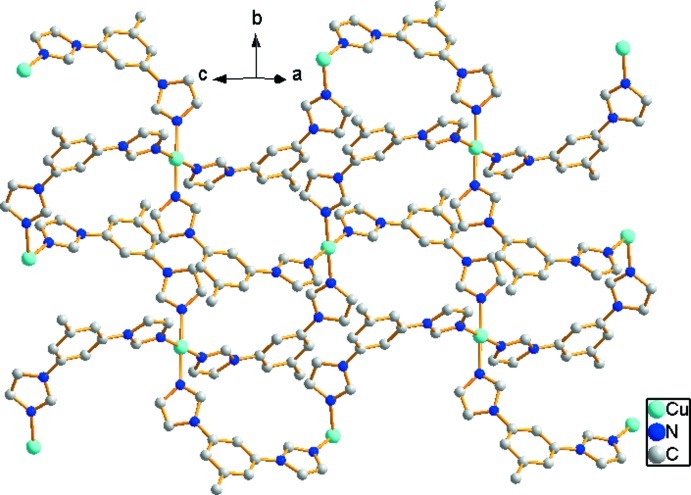
Lateral view of the two-dimensional sheet in the bc plane of the title complex. Hydrogen atoms, water molecules and nitrate anions are omitted for clarity.

**Fig. 3.**
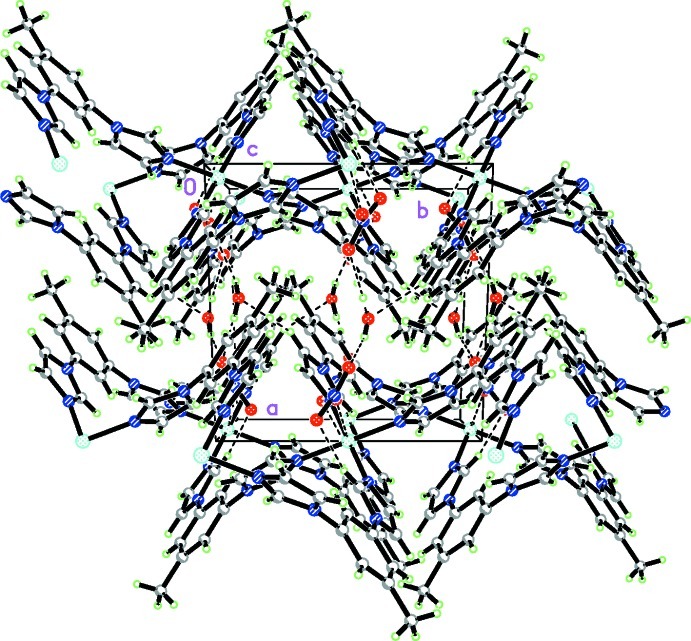
The packing diagram of the title complex, showing the hydrogen bonding as dashed lines.

## supplementary crystallographic information

### Comment

Imidazole has been well used in crystal engineering, and some zeolite-like porous frameworks with divalent and tetrahedral metal ions with this ligand have been reported (Huang *et al.*, 2006; Wang *et al.*, 2008; Tian *et al.*, 2007). Meanwhile, a large number of imidazole-containing flexible ligands have been extensively studied, and many fascinating coordination polymers based on such poly(imidazole) ligands have been synthesized (Jin *et al.*, 2008). However, to the best of our knowledge, the research on imidazole ligands bearing rigid spacers is still less developed (Qi *et al.*, 2008; Li *et al.*, 2007; Zhang *et al*., 2008).

Single-crystal X-ray diffraction analysis reveals that the title compound (I) crystallizes in the monoclinic space group *P*2_1_/*c*. The geometry of the Cu^II^ ion is surrounded by four imidazole rings of distinct *L* ligands and two nitrate anions, which illustrates a slightly distorted octahedral coordination environment (Fig. 1). Notably, as shown in Fig. 2, the four-coordinated Cu^II^ center is connected by the bent ligand *L* into a two-dimensional sheets in the *bc* plane. Within the ligand, the dihedral angle between the central benzene ring and terminal imidazole ring is 4.93 (11) and 46.08 (12), respectively. These sheets are further bridged into a three-dimensional supramolecular structure by O—H···O and C—H···O hydrogen bonds (Fig. 3).

### Experimental

The ligand was obtained according to the reported procedure (Altman *et al*., 2006). A mixture of CH_3_OH and H_2_O (1:1, 8 ml), as a buffer layer, was carefully layered over a solution of Cu(NO_3_)_2_ (0.02 mmol) in H_2_O (6 ml). Then a solution of 5-methyl-1,3-bis(imidazol-1-yl)benzene (*L*, 0.06 mmol) in CH_3_OH (6 ml) was layered over the buffer layer, and the resultant reaction was left to stand at room temperature. After two weeks, blue blocks of (I) appeared at the boundary. Yield: ~40% (based on *L*).

### Refinement

H atoms were positioned geometrically, with C—H = 0.93 and 0.96 Å for aromatic and methyl H atoms, respectively, and constrained to ride on their parent atoms, with *U*_iso_(H) = xUeq(C), where *x* = 1.5 for methyl H and *x* = 1.2 for aromatic H atoms.

### Crystal data

**Table:**

[Cu(NO_3_)_2_(C_13_H_12_N_4_)_2_]·2H_2_O	*F*(000) = 694
*M**_r_* = 672.12	*D*_x_ = 1.551 Mg m^−^^3^
Monoclinic, *P*2_1_/*c*	Mo *K*α radiation, λ = 0.71073 Å
Hall symbol: -P 2ybc	Cell parameters from 4382 reflections
*a* = 11.585 (4) Å	θ = 2.6–26.1°
*b* = 9.652 (3) Å	µ = 0.83 mm^−^^1^
*c* = 15.450 (4) Å	*T* = 293 K
β = 123.604 (17)°	Block, blue
*V* = 1438.9 (8) Å^3^	0.22 × 0.20 × 0.18 mm
*Z* = 2	

### Data collection

**Table:**

Bruker SMART APEX CCD area-detector diffractometer	2672 independent reflections
Radiation source: sealed tube	2114 reflections with *I* > 2σ(*I*)
Graphite monochromator	*R*_int_ = 0.039
phi and ω scans	θ_max_ = 25.5°, θ_min_ = 2.6°
Absorption correction: multi-scan (*SADABS*; Bruker, 2000)	*h* = −13→14
*T*_min_ = 0.839, *T*_max_ = 0.865	*k* = −11→11
10260 measured reflections	*l* = −18→18

### Refinement

**Table:**

Refinement on *F*^2^	Primary atom site location: structure-invariant direct methods
Least-squares matrix: full	Secondary atom site location: difference Fourier map
*R*[*F*^2^ > 2σ(*F*^2^)] = 0.036	Hydrogen site location: inferred from neighbouring sites
*wR*(*F*^2^) = 0.101	H atoms treated by a mixture of independent and constrained refinement
*S* = 1.06	*w* = 1/[σ^2^(*F*_o_^2^) + (0.0393*P*)^2^ + 1.4695*P*] where *P* = (*F*_o_^2^ + 2*F*_c_^2^)/3
2672 reflections	(Δ/σ)_max_ = 0.002
214 parameters	Δρ_max_ = 0.43 e Å^−^^3^
2 restraints	Δρ_min_ = −0.51 e Å^−^^3^

### Special details

**Table:**

Geometry. All e.s.d.'s (except the e.s.d. in the dihedral angle between two l.s. planes) are estimated using the full covariance matrix. The cell e.s.d.'s are taken into account individually in the estimation of e.s.d.'s in distances, angles and torsion angles; correlations between e.s.d.'s in cell parameters are only used when they are defined by crystal symmetry. An approximate (isotropic) treatment of cell e.s.d.'s is used for estimating e.s.d.'s involving l.s. planes.
Refinement. Refinement of *F*^2^ against ALL reflections. The weighted *R*-factor *wR* and goodness of fit *S* are based on *F*^2^, conventional *R*-factors *R* are based on *F*, with *F* set to zero for negative *F*^2^. The threshold expression of *F*^2^ > σ(*F*^2^) is used only for calculating *R*-factors(gt) *etc*. and is not relevant to the choice of reflections for refinement. *R*-factors based on *F*^2^ are statistically about twice as large as those based on *F*, and *R*- factors based on ALL data will be even larger.

### Fractional atomic coordinates and isotropic or equivalent isotropic displacement parameters (Å^2^)

**Table:**

	*x*	*y*	*z*	*U*_iso_*/*U*_eq_	
Cu1	0.0000	1.0000	0.5000	0.02941 (15)	
N1	0.1444 (2)	0.9308 (2)	0.64001 (17)	0.0311 (5)	
N2	0.2629 (2)	0.9008 (2)	0.80892 (16)	0.0283 (5)	
N3	0.1879 (2)	1.1172 (2)	1.05555 (16)	0.0310 (5)	
N4	0.0729 (2)	1.3057 (2)	1.04231 (17)	0.0333 (5)	
N5	0.1839 (3)	0.9479 (3)	0.3683 (2)	0.0465 (6)	
O1	0.1000 (3)	0.8849 (3)	0.28647 (19)	0.0649 (7)	
O2	0.1663 (2)	0.9523 (3)	0.44070 (18)	0.0589 (6)	
O3	0.2867 (4)	1.0002 (4)	0.3790 (3)	0.1209 (15)	
O1W	0.5399 (4)	0.9268 (4)	0.3928 (3)	0.0902 (10)	
C1	0.2547 (3)	0.8473 (3)	0.6687 (2)	0.0422 (7)	
H1	0.2756	0.8095	0.6235	0.051*	
C2	0.3288 (3)	0.8274 (3)	0.7721 (2)	0.0432 (7)	
H2	0.4089	0.7745	0.8110	0.052*	
C3	0.1522 (3)	0.9612 (3)	0.7259 (2)	0.0350 (6)	
H3	0.0892	1.0172	0.7290	0.042*	
C4	0.3023 (3)	0.9164 (3)	0.91399 (19)	0.0280 (6)	
C5	0.4142 (3)	0.8449 (3)	0.9937 (2)	0.0316 (6)	
H5	0.4639	0.7847	0.9791	0.038*	
C6	0.4530 (3)	0.8625 (3)	1.0961 (2)	0.0309 (6)	
C7	0.3782 (3)	0.9530 (3)	1.1169 (2)	0.0310 (6)	
H7	0.4033	0.9667	1.1848	0.037*	
C8	0.2661 (3)	1.0225 (2)	1.0358 (2)	0.0297 (6)	
C9	0.2265 (3)	1.0052 (3)	0.9345 (2)	0.0305 (6)	
H9	0.1502	1.0523	0.8808	0.037*	
C10	0.5755 (3)	0.7855 (3)	1.1834 (2)	0.0436 (7)	
H10A	0.6186	0.8405	1.2455	0.065*	
H10B	0.6411	0.7676	1.1652	0.065*	
H10C	0.5449	0.6993	1.1951	0.065*	
C11	0.1473 (3)	1.2442 (3)	1.0130 (2)	0.0359 (6)	
H11	0.1689	1.2832	0.9686	0.043*	
C12	0.0659 (3)	1.2134 (3)	1.1069 (2)	0.0372 (6)	
H12	0.0194	1.2287	1.1395	0.045*	
C13	0.1364 (3)	1.0975 (3)	1.1159 (2)	0.0363 (6)	
H13	0.1478	1.0195	1.1553	0.044*	
H1WA	0.469 (3)	0.958 (4)	0.393 (3)	0.071 (14)*	
H1WB	0.598 (5)	0.964 (5)	0.452 (2)	0.108 (19)*	

### Atomic displacement parameters (Å^2^)

**Table:**

	*U*^11^	*U*^22^	*U*^33^	*U*^12^	*U*^13^	*U*^23^
Cu1	0.0332 (3)	0.0317 (3)	0.0254 (3)	−0.0040 (2)	0.0176 (2)	−0.00066 (19)
N1	0.0347 (12)	0.0316 (12)	0.0307 (12)	0.0004 (10)	0.0205 (10)	−0.0015 (10)
N2	0.0314 (12)	0.0295 (11)	0.0269 (11)	0.0031 (9)	0.0180 (10)	0.0017 (9)
N3	0.0381 (12)	0.0306 (12)	0.0301 (12)	0.0057 (10)	0.0225 (11)	0.0020 (9)
N4	0.0379 (13)	0.0343 (12)	0.0307 (12)	0.0047 (10)	0.0208 (11)	0.0004 (10)
N5	0.0402 (14)	0.0553 (16)	0.0518 (17)	0.0045 (13)	0.0302 (14)	0.0115 (14)
O1	0.0625 (16)	0.0795 (18)	0.0558 (15)	0.0080 (14)	0.0348 (14)	−0.0128 (14)
O2	0.0598 (15)	0.0764 (16)	0.0548 (15)	0.0019 (12)	0.0408 (13)	−0.0031 (12)
O3	0.087 (2)	0.180 (4)	0.112 (3)	−0.049 (2)	0.066 (2)	0.009 (2)
O1W	0.089 (3)	0.090 (2)	0.088 (3)	−0.026 (2)	0.047 (2)	−0.026 (2)
C1	0.0524 (18)	0.0464 (17)	0.0377 (16)	0.0166 (14)	0.0311 (15)	0.0048 (13)
C2	0.0470 (17)	0.0500 (18)	0.0392 (17)	0.0212 (14)	0.0280 (15)	0.0076 (14)
C3	0.0363 (15)	0.0385 (15)	0.0329 (15)	0.0069 (12)	0.0209 (13)	−0.0012 (12)
C4	0.0312 (13)	0.0284 (13)	0.0277 (13)	−0.0014 (11)	0.0184 (11)	0.0007 (10)
C5	0.0344 (14)	0.0268 (13)	0.0376 (15)	0.0031 (11)	0.0224 (13)	0.0007 (11)
C6	0.0321 (14)	0.0263 (13)	0.0328 (14)	−0.0008 (11)	0.0169 (12)	0.0026 (11)
C7	0.0374 (15)	0.0301 (13)	0.0264 (14)	−0.0017 (11)	0.0182 (12)	0.0015 (11)
C8	0.0355 (14)	0.0277 (14)	0.0315 (14)	0.0009 (11)	0.0221 (12)	0.0001 (10)
C9	0.0315 (13)	0.0314 (13)	0.0287 (14)	0.0056 (11)	0.0169 (11)	0.0045 (11)
C10	0.0428 (17)	0.0446 (17)	0.0368 (16)	0.0118 (14)	0.0179 (14)	0.0093 (13)
C11	0.0491 (17)	0.0350 (15)	0.0331 (15)	0.0073 (13)	0.0287 (14)	0.0060 (12)
C12	0.0446 (16)	0.0406 (16)	0.0371 (16)	0.0016 (13)	0.0294 (14)	0.0000 (12)
C13	0.0504 (17)	0.0342 (15)	0.0360 (15)	0.0020 (12)	0.0312 (14)	0.0049 (12)

### Geometric parameters (Å, º)

**Table:**

Cu1—N1	1.980 (2)	C1—H1	0.9300
Cu1—N1^i^	1.980 (2)	C2—H2	0.9300
Cu1—N4^ii^	2.011 (2)	C3—H3	0.9300
Cu1—N4^iii^	2.011 (2)	C4—C5	1.381 (4)
N1—C3	1.313 (3)	C4—C9	1.383 (4)
N1—C1	1.360 (4)	C5—C6	1.395 (4)
N2—C3	1.345 (3)	C5—H5	0.9300
N2—C2	1.375 (3)	C6—C7	1.388 (4)
N2—C4	1.430 (3)	C6—C10	1.506 (4)
N3—C11	1.346 (3)	C7—C8	1.380 (4)
N3—C13	1.371 (3)	C7—H7	0.9300
N3—C8	1.434 (3)	C8—C9	1.376 (4)
N4—C11	1.316 (3)	C9—H9	0.9300
N4—C12	1.374 (3)	C10—H10A	0.9600
N4—Cu1^iv^	2.011 (2)	C10—H10B	0.9600
N5—O3	1.217 (4)	C10—H10C	0.9600
N5—O2	1.244 (3)	C11—H11	0.9300
N5—O1	1.246 (4)	C12—C13	1.346 (4)
O1W—H1WA	0.877 (19)	C12—H12	0.9300
O1W—H1WB	0.86 (2)	C13—H13	0.9300
C1—C2	1.344 (4)		
			
N1—Cu1—N1^i^	180.0	C5—C4—N2	120.8 (2)
N1—Cu1—N4^ii^	90.60 (9)	C9—C4—N2	118.7 (2)
N1^i^—Cu1—N4^ii^	89.40 (9)	C4—C5—C6	120.3 (2)
N1—Cu1—N4^iii^	89.40 (9)	C4—C5—H5	119.8
N1^i^—Cu1—N4^iii^	90.60 (9)	C6—C5—H5	119.8
N4^ii^—Cu1—N4^iii^	180.0	C7—C6—C5	119.3 (2)
C3—N1—C1	106.1 (2)	C7—C6—C10	120.2 (2)
C3—N1—Cu1	124.95 (19)	C5—C6—C10	120.5 (2)
C1—N1—Cu1	128.98 (18)	C8—C7—C6	119.3 (2)
C3—N2—C2	106.5 (2)	C8—C7—H7	120.3
C3—N2—C4	125.0 (2)	C6—C7—H7	120.3
C2—N2—C4	128.5 (2)	C9—C8—C7	121.8 (2)
C11—N3—C13	107.0 (2)	C9—C8—N3	117.8 (2)
C11—N3—C8	124.6 (2)	C7—C8—N3	120.3 (2)
C13—N3—C8	128.3 (2)	C8—C9—C4	118.8 (2)
C11—N4—C12	105.7 (2)	C8—C9—H9	120.6
C11—N4—Cu1^iv^	123.13 (18)	C4—C9—H9	120.6
C12—N4—Cu1^iv^	131.13 (18)	C6—C10—H10A	109.5
O3—N5—O2	119.9 (3)	C6—C10—H10B	109.5
O3—N5—O1	119.7 (3)	H10A—C10—H10B	109.5
O2—N5—O1	120.3 (3)	C6—C10—H10C	109.5
H1WA—O1W—H1WB	92 (4)	H10A—C10—H10C	109.5
C2—C1—N1	109.8 (2)	H10B—C10—H10C	109.5
C2—C1—H1	125.1	N4—C11—N3	111.2 (2)
N1—C1—H1	125.1	N4—C11—H11	124.4
C1—C2—N2	106.4 (2)	N3—C11—H11	124.4
C1—C2—H2	126.8	C13—C12—N4	109.8 (2)
N2—C2—H2	126.8	C13—C12—H12	125.1
N1—C3—N2	111.3 (2)	N4—C12—H12	125.1
N1—C3—H3	124.4	C12—C13—N3	106.3 (2)
N2—C3—H3	124.4	C12—C13—H13	126.8
C5—C4—C9	120.5 (2)	N3—C13—H13	126.8
			
N1^i^—Cu1—N1—C3	−12 (3)	C4—C5—C6—C10	−179.6 (2)
N4^ii^—Cu1—N1—C3	66.7 (2)	C5—C6—C7—C8	0.7 (4)
N4^iii^—Cu1—N1—C3	−113.3 (2)	C10—C6—C7—C8	−179.9 (3)
N1^i^—Cu1—N1—C1	170 (3)	C6—C7—C8—C9	−0.3 (4)
N4^ii^—Cu1—N1—C1	−111.9 (3)	C6—C7—C8—N3	−179.6 (2)
N4^iii^—Cu1—N1—C1	68.1 (3)	C11—N3—C8—C9	−44.8 (4)
C3—N1—C1—C2	0.0 (3)	C13—N3—C8—C9	132.7 (3)
Cu1—N1—C1—C2	178.8 (2)	C11—N3—C8—C7	134.5 (3)
N1—C1—C2—N2	0.0 (4)	C13—N3—C8—C7	−48.0 (4)
C3—N2—C2—C1	0.1 (3)	C7—C8—C9—C4	−0.6 (4)
C4—N2—C2—C1	−178.3 (3)	N3—C8—C9—C4	178.7 (2)
C1—N1—C3—N2	0.1 (3)	C5—C4—C9—C8	1.1 (4)
Cu1—N1—C3—N2	−178.82 (17)	N2—C4—C9—C8	−178.6 (2)
C2—N2—C3—N1	−0.1 (3)	C12—N4—C11—N3	0.0 (3)
C4—N2—C3—N1	178.4 (2)	Cu1^iv^—N4—C11—N3	177.32 (17)
C3—N2—C4—C5	176.8 (2)	C13—N3—C11—N4	−0.2 (3)
C2—N2—C4—C5	−5.1 (4)	C8—N3—C11—N4	177.7 (2)
C3—N2—C4—C9	−3.5 (4)	C11—N4—C12—C13	0.2 (3)
C2—N2—C4—C9	174.6 (3)	Cu1^iv^—N4—C12—C13	−176.8 (2)
C9—C4—C5—C6	−0.7 (4)	N4—C12—C13—N3	−0.3 (3)
N2—C4—C5—C6	179.0 (2)	C11—N3—C13—C12	0.3 (3)
C4—C5—C6—C7	−0.2 (4)	C8—N3—C13—C12	−177.5 (3)

Symmetry codes: (i) −*x*, −*y*+2, −*z*+1; (ii) *x*, −*y*+5/2, *z*−1/2; (iii) −*x*, *y*−1/2, −*z*+3/2; (iv) −*x*, *y*+1/2, −*z*+3/2.

### Hydrogen-bond geometry (Å, º)

**Table:**

*D*—H···*A*	*D*—H	H···*A*	*D*···*A*	*D*—H···*A*
O1*W*—H1*WA*···O3	0.88 (2)	2.04 (2)	2.909 (6)	170 (4)
O1*W*—H1*WB*···O3^v^	0.86 (2)	2.20 (3)	3.020 (5)	159 (5)
O1*W*—H1*WB*···O2^v^	0.86 (2)	2.42 (4)	3.142 (4)	142 (5)
C2—H2···O1*W*^vi^	0.93	2.36	3.230 (5)	156
C3—H3···O1^i^	0.93	2.27	3.186 (4)	167

Symmetry codes: (i) −*x*, −*y*+2, −*z*+1; (v) −*x*+1, −*y*+2, −*z*+1; (vi) *x*, −*y*+3/2, *z*+1/2.
